# Angiogenesis, Cardiomyocyte Proliferation and Anti-Fibrotic Effects Underlie Structural Preservation Post-Infarction by Intramyocardially-Injected Cardiospheres

**DOI:** 10.1371/journal.pone.0088590

**Published:** 2014-02-18

**Authors:** Eleni Tseliou, Geoffrey de Couto, John Terrovitis, Baiming Sun, Liu Weixin, Linda Marbán, Eduardo Marbán

**Affiliations:** 1 Heart Institute, Cedars-Sinai Medical Center, Los Angeles, California, United States of America; 2 Third Department of Cardiology, University of Athens, Athens, Greece; Albert Einstein College of Medicine, United States of America

## Abstract

**Objective:**

We sought to understand the cellular and tissue-level changes underlying the attenuation of adverse remodeling by cardiosphere transplantation in acute myocardial infarction (MI).

**Background:**

Cardiospheres (CSps) are heart-derived multicellular clusters rich in stemness and capable of multilineage differentiation. Post-MI CSp transplantation improves left ventricular (LV) function and attenuates remodeling in both small and large animal studies. However, the mechanisms of benefit have not yet been fully elucidated.

**Methods:**

Four groups were studied: 1) “Sham” (Wistar Kyoto rats with thoracotomy and ligature without infarction); 2) “MI” (proximal LAD ligation with peri-infarct injection of vehicle); 3) “MI+CSp” (MI with cardiospheres injected in the peri-infarct area); 4) “Small MI” (mid-LAD ligation only).

**Results:**

*In vivo* 1 week after CSp transplantation, LV functional improvement was associated with an increase in cardiomyocyte proliferation. By 3 weeks, microvessel formation was enhanced, while cardiomyocyte hypertrophy and regional fibrosis were attenuated. Collagen deposition was reduced, collagen degradation was enhanced, and MMPs were upregulated. The beneficial effects of CSp transplantation were not observed in the Small MI group, indicating that the effects are not solely due to CSp-induced cardioprotection. *In vitro*, CSp-conditioned media reduced collagen production in coculture with fibroblasts and triggered neoangiogenesis in an *ex vivo* aortic ring assay.

**Conclusion:**

Cardiospheres enhance cardiomyocyte proliferation and angiogenesis, and attenuate hypertrophy and fibrosis, in the ischemic myocardium. These synergistic effects underlie the attenuation of adverse remodeling by cardiospheres.

## Introduction

Following MI, the architecture and function of the left ventricle (LV) becomes distorted in a process known as ventricular remodeling [1]. Treatment approaches have focused on limiting the initial injury by prompt reperfusion, followed by blocking secondary maladaptive pathways; however, ventricular remodeling not infrequently ensues even with best current practices [2]. Cell therapy seeks, instead, to regrow the lost myocardium and restore cardiac function [3]–[5]. Notable among cell products currently in the process of clinical translation [6]–[10] are “cardiospheres”: heart-derived self-assembling three-dimensional multicellular clusters which express stem cell markers and are capable of multilineage differentiation, although paracrine mechanisms seem to predominate *in vivo* [11]–[18].

While several laboratories have shown functional benefits with cardiosphere transplantation [19]–[25], a detailed understanding of the underlying tissue-level changes is essential to facilitate therapeutic applications. What are the effects of cardiosphere transplantation on angiogenesis? Is post-MI cardiomyocyte hypertrophy blunted? Can the beneficial effects of cardiospheres be fully explained by tissue preservation and infarct size reduction in the acute ischemic setting? Do cardiospheres inhibit the formation of scar tissue? Are favorable changes in the peri-infarct area also evident in remote non-ischemic myocardium? Here, we addressed these questions with a view to defining the structural changes that underlie the ability of CSps to attenuate post-MI ventricular remodeling.

## Methods

Four groups of female Wistar-Kyoto rats were studied: 1) “Sham” operated animals implanted with a stitch around the proximal left anterior descending artery (LAD) without ligation [12]; 2) “MI” group with a permanent proximal LAD ligation followed by peri-infarct injection of vehicle only (placebo); 3) “MI+CSp” with MI induced as in group 2 and cardiospheres injected in the peri-infarct area (at a dose of 2M syngeneic CDCs [12], [13], CSp-treated group); and 4) “Small MI” group with more distal, mid-LAD ligation, designed to mimic the reduced infarct size seen in group 3. For clarity, results from groups 1–3 are presented initially, with comparison later to group 4 for mechanistic purposes. However, all experiments were performed contemporaneously.

### Ethics statement

The study was approved by the Institutional Review Board of Cedars Sinai Medical Center (2424/2008). All animal procedures were conducted in accordance with humane animal care standards outlined in the NIH Guide for the Care and Use of Experimental Animals and were approved by the Cedars-Sinai Medical Center Animal Care and Use Committee. Human cardiac derived stem cells were obtained from written informed and written consenting patients, and the tissue harvesting was approved by Institutional Review Board of Cedars Sinai Medical Center (14713). Human dermal fibroblast cell lines were purchased by Promocell GmbH (Germany).

### Surgery and Echocardiography study

Secondary cardiospheres were derived from male Wistar Kyoto rats (4–6 wks old) as described [11], [26]. These cells have been shown to express stem cell markers such as c-kit in their core and CD105 on their periphery and maintain a cardiomyogenic differentiation phenotype [11], [26]. Myocardial infarction with permanent LAD ligation was induced in Female Wistar Kyoto rats (6–8 wks old). Shortly afterwards, 2M CDCs in cardiospheres resuspended in 120 µl of PBS or 120 µl of PBS were injected in four sites in the peri infarct area. All surgery was performed according to the ARRIVE guidelines under isoflurane anesthesia, and all efforts were made to minimize suffering.

Two-dimensional echocardiograms and targeted M-mode echocardiograms were obtained using the Vevo770 (VisualSonics, Toronto, ON, Canada) at baseline (18–24 h post surgery), at day 7 and day 21 as described [12]. Briefly, all animals were mildly anesthetized with 2% inhaled isoflurane and then two-dimensional short axis views of the LV were obtained at the level of papillary muscles. All echocardiographic data represent the mean of three measurements in different cardiac cycles. The following parameters were evaluated: LV ejection fraction (EF), LV end diastolic diameter (LVEDD), and LV posterior wall thickness (LVPWT). Six animals (n = 6) were analyzed in each group at each time point.

### Tissue processing and staining

For histological analysis, five animals per group were sacrificed at 1 and five animals at 3 weeks after myocardial infarction and CSp transplantation. Hearts arrested in diastole were excised, embedded in Tissue-Tek OCT compound (Sakura, Torrance, California) and kept in −80°C until sectioning. 5 µm sections were cut and processed accordingly. For LV morphometry evaluation, 5–10 slides per heart were stained with Masson's trichrome (Sigma) and quantification of the infarct size, infarct mass, and viable mass were performed as previously described [12], [13] using Image J software.

Cardiosphere-induced host cardiomyocyte proliferation was evaluated by Ki67 staining. Tissue sections at day 7 post MI and treatment were fixed with 4% PFA and incubated overnight with Rabbit anti-rat ki67 (Abcam, Cambridge, Massachusetts) and mouse anti-rat α-sarcomeric actin (Sigma Aldrich, St. Louis, Missouri) followed by proper secondary antibodies and DAPI (4′,6-diamidino-2-phenylindole). The ratios of proliferating cardiomyocytes to the total number of cardiomyocytes and the total number of cells per field were evaluated at both peri-infarct and remote zones. Five different heart tissues per group and approximately 10–15 different high-power images per animal were examined and the average was used for statistical analysis. One hundred cardiomyocytes per heart were evaluated.

For cardiomyocyte cross-sectional area evaluation, 3–5 sections of 5 µm thickness per heart were stained with Wheat Germ Agglutinin (Invitrogen, Carlsbad, California), α-sarcomeric actin (Sigma) and DAPI. Only myocytes in which the nucleus was centrally located within the cell were analyzed. Approximately 10–15 high-power fields per area from a minimum 3 slides per heart were analyzed to obtain the average regional cross-sectional area and regional myocyte nuclear density. One hundred cardiomyocytes per heart were evaluated.

Vascular density was quantified in animals sacrificed 3 weeks post MI. For capillary density (capillaries/field) a total of 3–5 sections per heart were stained with Rabbit anti- rat von Willebrand Factor antibody (vWf, Abcam) and 10–15 images from the peri-infarct and remote area per section were evaluated. Both small and large capillaries were evaluated: all capillaries with more than four vWf positive nuclei were recorded. Arteriolar density (arterioles/field) was quantified in a similar way after staining with mouse anti-rat α-smooth muscle actin (sma, Abcam) separate sections. All immunohistochemistry pictures were taken on a laser scan confocal microscope (Carl Zeiss Microscopy, Munich, Germany).

For Sirius red staining, 3–5 serial sections per heart from the base to apex were immersed in Sirius red (Sigma) for 60 min, differentiated 2 min in Hcl 0.01N, dehydrated, and mounted in resin mounting media (Electron Microscopy Sciences, Hatfield, Philadelphia). The degree of fibrosis was detected by quantification of collagen deposition in the infarct site, peri infarct, and remote zone separately. Approximately, 15–20 high-power images under polarized light in each zone were evaluated. Fibrosis was measured as percent of collagen area (red) versus total tissue area (black).

### Conditioned media isolation

Human CSps were cultured and quantified as described [11]. In brief, CDCs were harvested and plated in ultra-low attachment dishes with serum free culture media. 48 hours later, CSps were harvested by mild aspiration and centrifuged at 1500 rpms for 5 min. The supernatant was further centrifuged at 400 g for 5 min and at 2000 g for 10 min in order dead cells and debris to be eliminated.

For the cocultures with human dermal fibroblasts, fibroblasts purchased from Promocell (Germany) [27] were serum depleted overnight. Supernatant from CSps was added in the fibroblasts for 24 additional hours in a 1∶1 ratio. Serum free media was used at all stages of this experiment.

For the purposes of the *ex vivo* angiogenesis assay, 10% FBS (Fetal Bovine Serum, Invitrogen, Carlsbad, California) media was used for the CSp formation and 10% FBS culture media was used as one of the controls. All *in vitro* experiments were performed in triplicate.

### *Ex vivo* angiogenesis assay

Female Wistar Kyoto (WK) rats of the same age as those used for the *in vivo* experiments were processed as described [28]. Aortic rings from the thoracic aorta were collected and cut in 1 mm long aortic rings. The rings were embedded in rat tail interstitial collagen matrice prepared by mixing collagen (Collagen R, SERVA Electrophoresis GmbH, Heidelberg, Germany), IMDM (Iscove's Modified Dulbecco's Medium, Invitrogen), and NaHCO_3_ (Sigma Aldrich) with pH adjusted to 7.4. After overnight serum-free starvation, CSp-conditioned media, 10% FBS (Invitrogen) culture media or specific endothelial basal media (Lonza, Walkersville, Maryland) was added. Media was changed every other day in all three groups up to total 9 days of treatment. The number of sprouts originating from the ring was evaluated at the last day under phase contrast microscopy. The CSp-conditioned media treated aortic ring was fixed and used for immunohistochemistry for further sprout evaluation. Mouse anti rat sma (Sigma Aldrich) and BS1 lectin (Sigma Aldrich) were the primary antibodies used. Three different experiments per group were analysed.

### Tissue Cytokine evaluation

LV tissue protein taken separately from the peri infarct and the remote myocardium at day 21 post MI (n = 3 in each group) was extracted and quantified as described [12], [13], [26]. 100 µg of total protein amount (n = 3 in each group) was used for the inflammatory cytokines IL-1b, TNF-a, IFN-g, and IL-6 measurement according to the manufacturer's instructions (RayBiotech, Norcross, Georgia). The results were analyzed by densitometry.

### Hydroxyproline quantification

Post-mortem LV tissue was isolated, visually dissected into three different regions (infarct site, border zone, and remote zone), and frozen in liquid nitrogen. Each tissue was then separately weighed, lyophilized, pulverized, and processed as described [5] (hydroxyproline assay kit, Biovision Inc., Milpitas, CA). More than 10 tissue samples per group were evaluated from the different heart regions. The results are represented as mg of hydroxyproline per g of dry tissue weight.

### Silencing MMP2 and MMP9 expression

Expression of MMP2 and MMP9 was silenced with a small interfering RNA (siRNA) sequence against human MMP2 and MMP9 (ON-TARGETplus SMARTpool siRNA sequences, Dharmacon, Thermo Scientific). Human CDCs were transfected in serum-free, antibiotic-free medium using Lipofectamine 2000 (Invitrogen) as the vehicle according to the manufacturer's protocol. After transfection, CDCs were plated for 48 h in ultra-low attachment dishes in order to form CSps. The knockdown efficiencies of RNAi were confirmed by determining the decreases in the level of MMP2 and MMP9 expression in the supernatant by zymography, and in CSps by RT-PCR (data not shown). According to this protocol a maximum gene silencing of 75% can be achieved. Prism 7900 sequence-detection system (Applied Biosystems, Foster City, California) for 60 total cycles was used. The experiment was performed in triplicate.

### Measurements of collagen metabolism

To evaluate collagen degradation, plasma samples were collected at day 7 after MI induction and the carboxyterminal telopeptide region of type I collagen enzyme-immunoassay kit (ICTP, Orion Diagnostica, Finland) was used according to manufacturer's instructions. More specifically, blood was collected before animal sacrifice. Samples were immediately centrifuged and the plasma layer was aliquoted and stored in −80°C avoiding repeated freezing and thawing cycles. For the final quantification the samples were thawed on ice. All measurements were performed in duplicate. More than ten samples in each group were used for this assay.

### Western Blots and zymograms

To evaluate the expression of MMPs and TIMPs, LV samples from the peri infarct zone where the CSps had been injected were homogenized and processed as previously described with small modifications [12], [13]. More specifically, LV tissues (n = 3 in each group) were homogenized in tissue lysis buffer (Thermo Scientific) without protease inhibitors and stored at −80°C. Final protein concentration was quantified with a standardized colorimetric assay (BCA Protein Assay, Thermo Scientific, Rockford, Illinois). Gelatin zymography was used to determine MMP2 and MMP9 pro form and active form while the rest of the MMPs and TIMPs of this study were evaluated by immunoblotting. Both zymograms and immunoblots were analyzed by densitometry Protein level in the dermal fibroblasts used in the in vitro cocultures was also determined by BCA Protein Assay (Thermo Scientific). Rb anti human col1 (Abcam) was used for the collagen detection.

### RNA isolation and semi-quantitative reverse transcriptase polymerase chain reaction for Coll1a1 and Coll3a1 expression

Although the collagen concentration of the myocardium can be obtained from a determination of the collagen-specific amino acid hydroxyproline, or the morphometric assessment of collagen fibers by Sirius red staining, neither technique enables the identification of the collagen phenotypes; for this purpose we performed RT-PCR for coll1a1 and coll3a1 (Applied Biosystems). RNA was isolated from LV samples from the peri-infarct and remote zones (n = 3 in each group) (RNA easy kit, Qiagen, Germantown, Maryland) and reverse-transcribed into cDNA according to manufacturer's protocol. PCR amplification was performed in 25 µl of reaction mixture. Relative mRNA expression of target genes was normalized to the endogenous Gapdh gene.

### CSp immunostaining

CSps were stained according to a described protocol [11], [26]. In brief, spheres were fixed in ethanol/acetone and stained in whole mount. The following primaries diluted in Dako saponin 0.1% (DAKO) were used: Rabbit anti human MMP2 (Abcam) and Rb anti human MMP9 (Abcam). Secondary antibodies conjugated with Alexa fluorochromes were used.

### Statistical Analysis

Student's t test was used to assess significance of group differences at p<0.05. Intergroup comparisons were made with one way ANOVA. Results are mean ±SEM unless otherwise reported. Data were analyzed using GraphPad Prism software (version 5.00, GraphPad, San Diego, California).

## Results

### LV architecture

**Figure 1:**
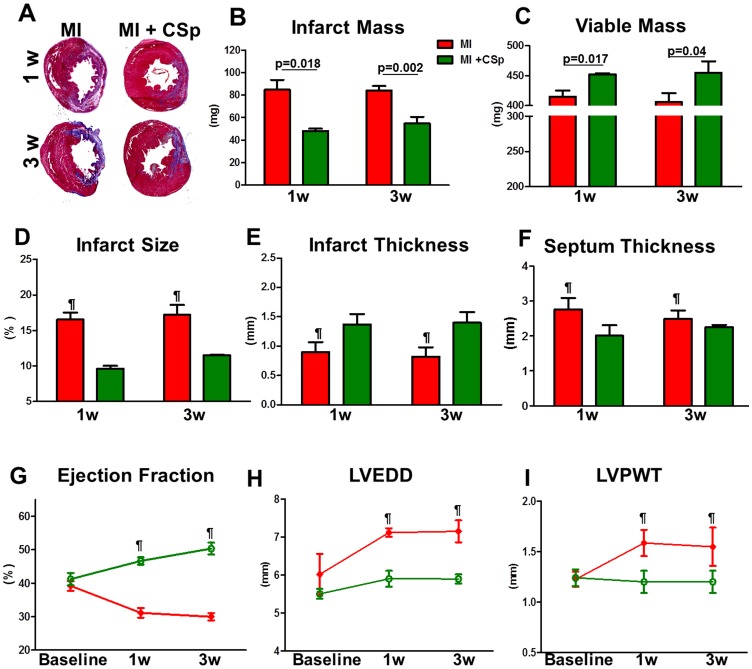
Tissue morphology and cardiac function. (A) Representative photomicrographs of myocardial sections stained with Masson's trichrome. Sections are from CSp treated (n = 5) and placebo treated (n = 5) animals at 7 and 21 days post MI and treatment. (B–F) Quantitative data for infarct mass, viable mass, infarct size, infarct thickness and septum thickness. Five different hearts were processed and 5–6 different sections from the apex to the base from each heart were used for the analysis. (G–I) EF ejection fraction, LVEDD left ventricular end diastolic diameter, LVPWT left ventricular posterior wall thickness in diastole. Baseline represents measurements 18–24 hours after coronary ligation and treatment. n = 6 for each animal group studied. Bars are presented as mean±SD. ¶ p<0.05 control vs MI +CSp.

We performed Masson's trichrome staining to evaluate infarct size one week and three weeks post-MI (Figure 1A). Five different hearts were processed and 5–10 different sections from the apex to the base from each heart were analyzed. Infarct size was defined as infarct mass divided by total LV mass. By three weeks post-MI, we found a scar mass reduction of 35%, while viable mass was increased by 13%, in the CSp-treated group (Figure 1B, 1C). Scar size in the MI+CSp group was significantly reduced relative to the MI group one week post treatment (Figure 1D). We also evaluated septal and infarct wall thickness in the same sections. At three weeks, CSp-treated hearts had a 20% lower septal thickness and a 35% higher infarcted wall thickness (Figure 1E, 1F). The increase in infarct wall thickness and the attenuated septal wall thickness, indicate relatively preserved cardiac tissue structure with cardiospheres evident as soon as 1 week post-MI.

### LV Dimensions and Function

There was no difference in the baseline echocardiographic parameters between the MI and the MI+CSp groups. However, LV ejection fraction (EF) at 1 and 3 weeks post-MI was greater in the MI+CSp group compared to the MI group (p<0.05; Figure 1G). In addition, LV dilation was attenuated (as revealed by lower LV end-diastolic diameter [LVEDD]; Figure 1H), while reduced compensatory hypertrophy was evidenced by lower LV posterior wall thickness (LVPWT) in diastole in CSp-transplanted hearts (Figure 1I).

### Cardiomyocyte size and proliferation

**Figure 2:**
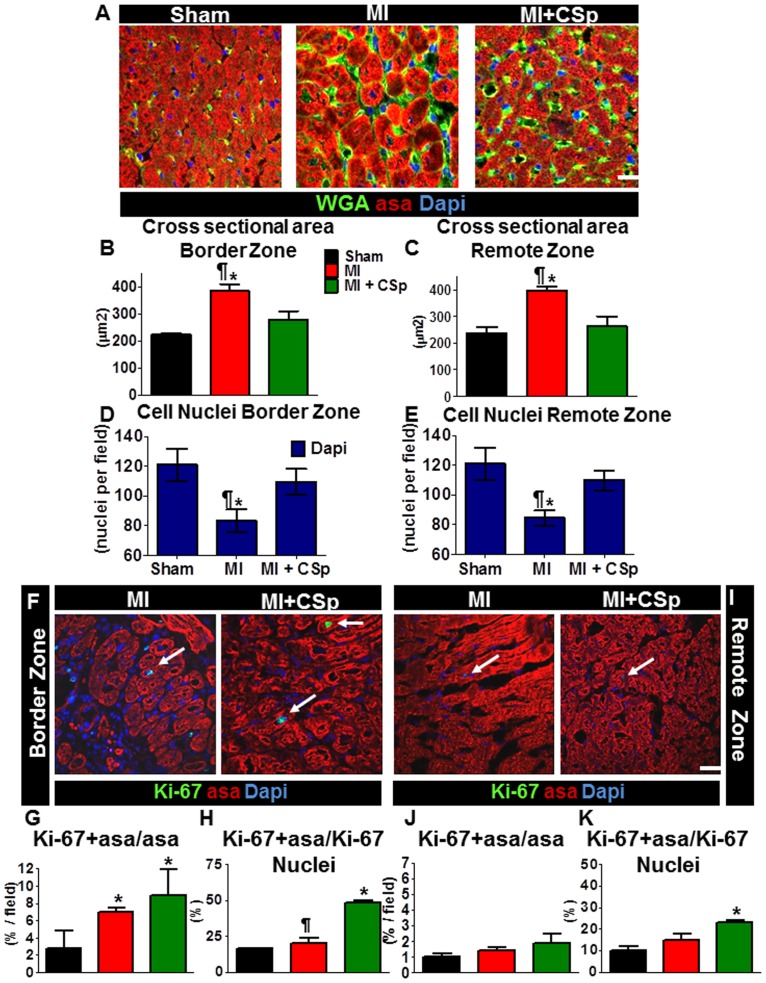
Cardiac tissue structure. (A) Representative photomicrographs of immunohistochemical staining of smooth muscle actin, wheat germ agglutinin, and Dapi in myocardial tissue sections. Five different heart tissues per group were stained. Approximately 10–15 high-power fields per area from a minimum 3 slides per heart were analyzed to obtain the average regional cross sectional area and regional myocyte nuclear density. 100 cardiomyocytes per heart were evaluated. (B) Quantification of cross-sectional area in the peri-infarct and D. in the remote zone respectively. (C,E) Measurement of the total number of cell nuclei per field evaluated in both above-mentioned regions. (F) Representative photomicrographs of immunohistochemical staining of α-sarcomeric actin, Ki67 and Dapi, in the peri-infarct zone and (I) in the remote area. (G–H) Quantification of proliferating cardiomyocytes per total number of cardiomyocytes per field and of proliferating cardiomyocytes per total nuclei per field respectively. (J–K) Same quantification as in G–H but in the remote area. Arrows point to Ki67+/asa+ cells. (Scale bars 50 µm). Data are mean±SEM. ¶ p<0.05 control vs. MI+CSp, * p<0.05 vs. sham.

A major compensatory mechanism post-MI is hypertrophy of surviving cardiomyocytes. We sought to determine whether the gross changes in viable mass reflect cardiomyocyte hypertrophy or increased myocyte number. Samples from 5 different hearts per group, 3 weeks post-MI were stained with alpha sarcomeric actin (a-sa), wheat germ agglutinin (wga, a stain for the surface cell membrane) and Ki67^+^ (a marker of active proliferation). The absolute number of cardiomyocytes was reduced in both the peri-infarct and remote zones of the MI group, and cross-sectional area was increased, indicating compensatory myocyte hypertrophy (Figures 2A–E). However, in the CSp-treated myocardium we found a ∼10% higher myocyte nuclear density and attenuated hypertrophy in both regions (Figures 2B–E). In addition, the number of α-sa^+^/Ki-67^+^ cells was markedly higher than in the placebo groups (Figures 2F–K). Thus, we conclude that the augmentation of viable mass in CSp-treated hearts (Figure 1C) reflects myocyte hyperplasia and/or myocyte preservation rather than hypertrophy.

### Tissue vascularity

**Figure 3:**
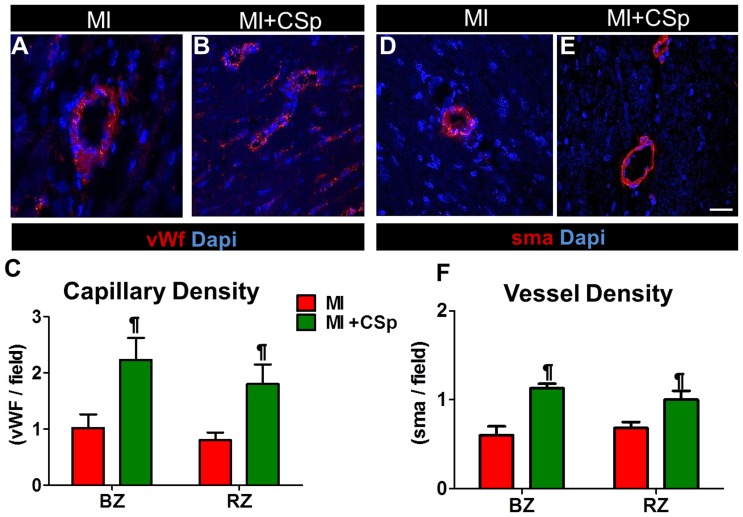
Cardiospheres stimulate angiogenesis. (A,B) Photomicrographs of vWf (red) stained capillaries and (D,E) smooth muscle actin (red) vessels in the border zone of CSp transplanted and vehicle treated animals. 10–15 images from the peri infarct and remote area per section were evaluated. For quantification both small and large capillaries were evaluated. (C) Quantification of the capillary density and (F) of the vessels' density in both border and remote regions. Significant increases in both structures were detected post CSp transplantation in both regions studied. Five different heart tissues per group were analyzed. Abbreviations. IZ (infarct zone), BZ (border zone), RZ (remote zone). (Scale bars 50 µm). Data are mean±SD. ¶ p<0.05 control vs. MI+CSp.

In order to evaluate the effect of CSps on the capillaries and on the vessels, we performed immunohistochemistry (n = 5 in each group) with vWf and sma respectively in tissue samples 3 weeks post-MI. Ten-fifteen images from the peri-infarct and remote areas were evaluated per section. Both capillaries and microvessels were quantified as total number per field (Figures 3A, 3B). Three weeks after CSp transplantation we found a ∼twofold-higher capillary density in the peri-infarct zone relative to placebo, with a similar but smaller trend in the remote area (Figures 3A–C). The density of microvessels (sma+) was also increased, especially in the peri-infarct area (representative images in Figures 3D,E; pooled data in Figure 3F). We have previously observed that engraftment of autologous or allogeneic CSps is very low 3 weeks after transplantation (<<1%) [26]. Thus, it is likely that paracrine mechanisms known to favor angiogenesis [25], [26] underlie the enhanced vascularity observed here. However, preservation of pre-existing vessels due to attenuated MI expansion cannot be excluded.

### *Ex vivo* angiogenesis assay

**Figure 4:**
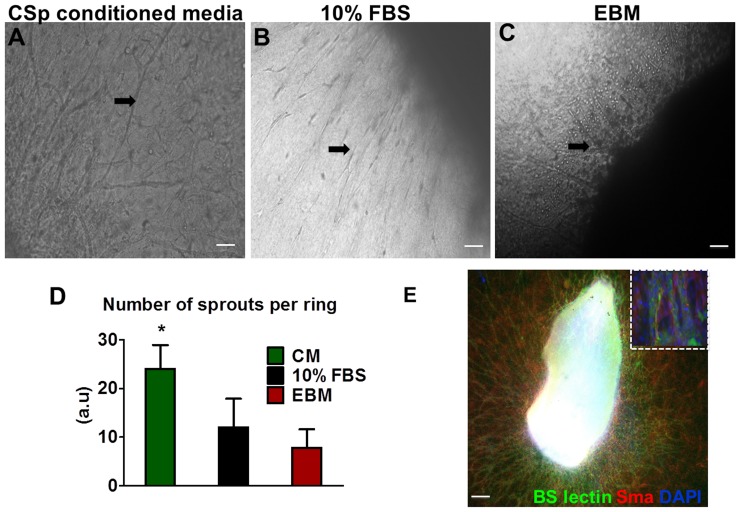
Aortic ring sprouts. Representative phase contrast images from aortic rings embedded in collagen matrices 9 days post treatment with (A) CSp conditioned media, (B) 10% FBS culture media and (C) endothelial basal media. (D) Quantification of sprouts on day 9 under phase contrast microscopy. Arrows point at the new sprouts. (E) Immunofluorescence of the CSp conditioned media treated aortic ring reveals the phenotype of the de novo formed microvessels. Inserted photo is a high power field image of the immunostained microvessels for BS-lectin and smooth muscle actin. Three different experiments per group were evaluated (Scale bars 10 µm). Data are mean±SD. ¶ p<0.05 control vs. MI+CSp.

To further verify a paracrine angiogenic effect of CSps, we performed an *ex vivo* angiogenesis assay [28] (Figure 4A–C). The number of *de novo* sprouts originating from the aortic ring was greatest in the CSp-conditioned media group 9 days post treatment compared to the other treated groups (p<0.05) (Figure 4D). In addition, fixed rings were further processed with immunohistochemistry to verify the endothelial and microvessel phenotype of the sprouts (Figure 4E). Since only CSp-conditioned media was used in this assay, the new vessel formation here arises purely from paracrine effects of CSps.

### Tissue inflammatory cytokines IL-1β, TNFα, IL-6 and IFNγ

**Figure 5:**
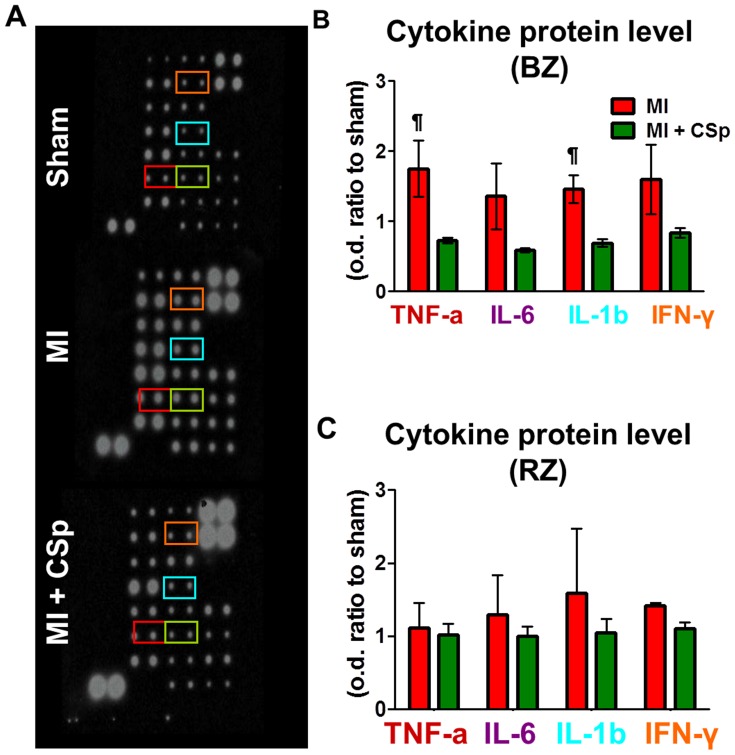
Proinflammatory cytokine expression. (A) Representative densitogram of cytokine expression in the peri infarct region 21 days post MI and CSp transplantation. (B, C). CSp treatment attenuated the inflammatory tissue cytokines' expression. IL-1b and TNF-α were significantly decreased in the peri-infarct region of the CSp transplanted myocardium while a trend was detected in the remote. Three different samples per group were analysed. Data are mean±SD. ¶ p<0.05 control vs. MI+CSp.

Post-MI inflammatory cytokines play an important role in myocardial remodeling [29]. Although beneficial at early stages post-MI, persistence of inflammatory cells initiates a vicious cycle of cytokine stress [29]. We examined tissue cytokine expression 3 weeks post-MI, a time when the remodeling process is largely complete in rodents [30], [31]. Figure 5A shows representative images from the cytokine protein array. We focused on IL-1β, TNF**α**, IL-6 and IFN**γ** which have been associated with the post-MI adverse remodeling and we found a decreased level in the peri-infarct zone of the CSp-treated hearts relative to placebo, reaching significance for IL-1β and TNF-**α** (Figure 5B). No statistically significant differences were evident in the remote zone (Figure 5C). These changes in the cytokine profile may be secondary to cardioprotection produced by transplanted CSps, given their known anti-inflammatory and immunomodulatory properties [26], recapitulated by other cardiac-derived cell preparations [32], [33].

### Sirius Red staining for collagen accumulation

**Figure 6:**
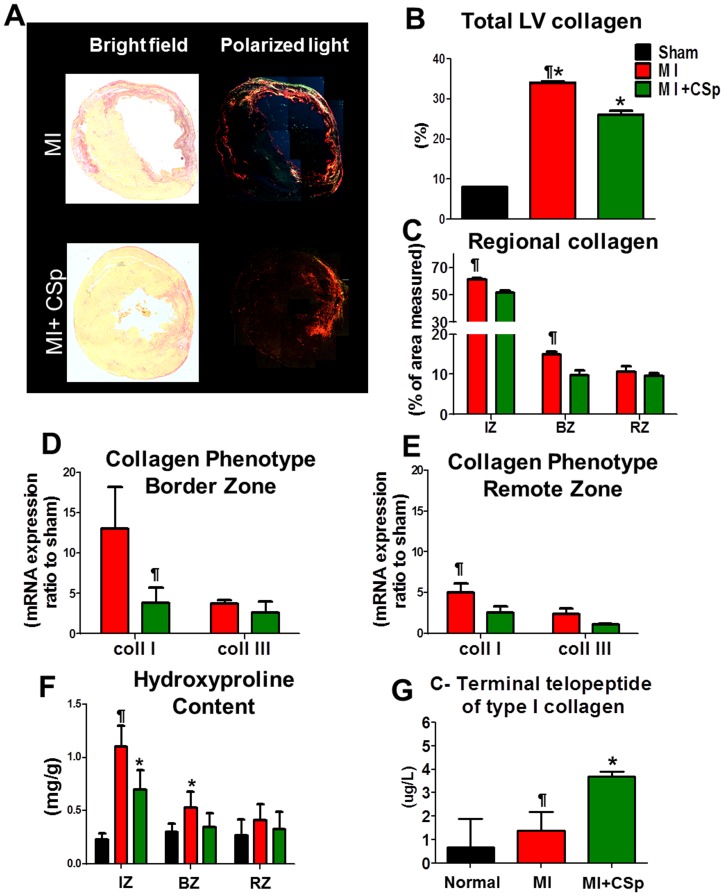
Collagen quantification. (A) Collagen deposition was detected by picrosirius red staining in tissue sections 21 days post MI and CSp transplantation. Representative tissue images under bright field on the left and under polarized light microscope on the right. (B) Quantification of the collagen in the total LV area and C. separately in the infarct, border and remote zone. (D,E) Results of RT-PCR for mRNA expression of coll1a1 and coll3a1 respectively. (F) Hydroxyproline assay performed for collagen content production in the infarct, border, and in the remote zone. Significant differences between the two groups were observed in the infarct and in the border areas. (G) ICTP quantification in the serum collected 7 days post MI and treatment with increased levels detected in the treated group. Abbreviations. IZ (infarct zone), BZ (border zone), RZ (remote zone). Data are mean±SEM. ¶ p<0.05 control vs. MI+CSp, * p<0.05 vs. sham.

The extracellular matrix (ECM) undergoes major changes that largely define the phenotype of ventricular remodeling [30], [31]. The deposition and degradation of fibrillar collagen skyrocket post-MI, with progressive net accumulation of collagen [30], [34]. Here we asked whether the CSp-related changes in scar mass manifested grossly are due to underlying changes in collagen content evaluated by Sirius Red staining (Figure 6A). Total LV collagen was lower in the CSp-treated hearts compared to placebo three weeks post-MI (Figure 6B). In addition, we detected a lower collagen density specifically in the peri-infarct and infarct regions, with a similar but non-significant trend in the remote myocardium (Figure 6C). These findings may reflect reduced collagen production by resident fibroblasts and/or increased collagen degradation by activated MMPs, which are highly expressed by cardiospheres [13]. The latter conjecture is tested in detail below.

### Biochemical quantification of collagen

An important characteristic of post-MI remodeled myocardium is the increased wall stiffness which promotes LV dilation and dysfunction [30], [35], [36]. Procollagen molecules are secreted into the ECM where they undergo serial processing to become mature structural collagen fibrils [36], increasing tissue stiffness. We performed RT-PCR for Collagen I and Collagen III expression in peri-infarct and remote myocardium 3 weeks post-CSp or PBS transplantation. The transcript-level expression of collagen I fibers in both regions was reduced in the CSp-treated compared to the control group (p<0.05) (Figures 6D, 6E). No differences in the expression of collagen III were detected in either the peri-infarct or remote zones. In addition, total hydroxyproline content remained unchanged in the remote zone, but was diminished in the infarct and border zones of CSp-treated hearts relative to control (Figure 6F). The reduced collagen deposition likely contributes to the improved functional and structural profile of CSp-treated myocardium.

### Markers of collagen turnover

To test the notion that CSps may exert a collagenolytic effect, we measured circulating levels of the C-terminal telopeptide of type I collagen (ICTP), a breakdown product of fibrillar collagen I (which accounts for over 80% of myocardial collagen [37]). For each collagen type I fiber produced, one ICTP molecule is released into the bloodstream [37]. Serum from day 7 post-MI was used and processed according to the manufacturer's instructions. Higher levels of plasma ICTP levels were detected in the CSp-treated group than in placebo (>50%) (Figure 6G), consistent with enhanced collagenolysis after CSp transplantation [38].

**Figure 7:**
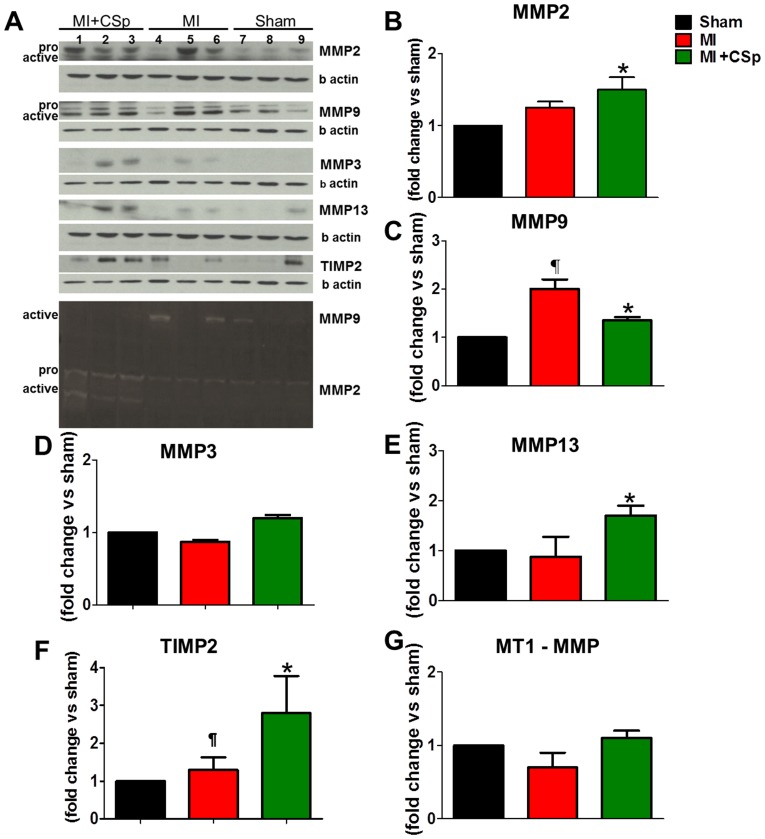
Metalloproteinase activity. (A) Immunoblotting (top 10) and zymograms (bottom membrane) were performed to examine the relative abundance of representative MMP types in myocardial tissue obtained from the peri-infarct zone 7 days post MI and CSp injection. For MMP2 and MMP13 the same membrane was used. For MMP9, MMP3 and TIMP2 the same membrane was used as well. The same samples were used in both membranes. All three samples in each group were evaluated. (B,E,F) Increases in MMP2, MMP13, and TIMP2 were observed in the peri-infarct zone of the post CSp transplanted myocardium. (C) In contrast, relative levels of MMP9 increased in the control compared to the treated group. (D,G) No differences were detected for MT1-MMP and MMP3. Data are mean±SD. ¶ p<0.05 control vs. MI+CSp, * p<0.05 vs. sham.

Other commonly-evaluated markers of collagen breakdown are the MMPs and their tissue inhibitors TIMPs; their balanced action defines whether ECM collagen is deposited or degraded [36]–[38]. We measured representative remodeling-associated [30], [38] members of the different MMP classes: the interstitial collagenase MMP13, the gelatinases MMP2 and MMP9, and MMP3 from the matrilysin subclass. We performed Western blots for the abovementioned MMPs, and zymograms for MMP2 and MMP9, 7 days post-MI (Figure 7A, Western Blots on top and Zymogram on the bottom). Active MMP2 and MMP13 levels were upregulated in the CSp-treated group compared to placebo, where only MMP9 was elevated (Figures 7B–E). No differences were seen in the other MMPs evaluated (Figures 7D, 7G). Notable is the upregulation of TIMP2 (Figure 7F), which has dual effects by both activating and deactivating MMP2 [38]. Our findings of increased active MMP2 indicate a collagenolytic property of CSps as early as 7 days post-MI, a time at which scar size is already reduced in CSp-treated hearts (Figure 1D). An unexpected finding was the increase in MMP9 in the placebo group. MMP9 upregulation in the long-term post-MI setting is associated with adverse ECM turnover, a characteristic of remodeled myocardium [39], [40]. Injected cardiospheres may either degrade collagen, or inhibit new collagen formation. Either possibility is consistent with the reduced hydroxyproline content detected in the treated group in both the border and in the infarct zone, while the higher serum ICTP levels support greater degradation.

### *In vitro* co-cultures of CSp-conditioned media and fibroblasts

CSps express high levels of MMPs such as MMP3, MMP13, MMP10 and MMP11 [13]. Here we showed that MMP2, and to a lesser extent MMP9, are also expressed (Figure S1A, 1B). To assess the potential paracrine collagenolytic effect of CSps, we performed an *in vitro* coculture experiment with CSp-conditioned media and human dermal fibroblasts (Figure S1C, 1D, 1E). Protein extracted from the fibroblasts was used for western blot analysis in order to evaluate the protein expression of collagen after exposure to CSp-conditioned media. Collagen I protein levels were reduced in CSp-conditioned media coculture relative to fibroblasts cultured conventionally (Figure S1F).

To further test whether MMP2 and MMP9 secreted by CSps are able to degrade collagen, we repeated the same coculture experiments after silencing MMP2 and MMP9 gene expression in CSps. SiRNA was applied according to the manufacturer's protocol (ON-TARGETplus SMARTpool siRNA sequences, Dharmacon, Thermo Scientific). Zymography for MMP2 and MMP9 confirmed muted protein expression in the CSp supernatant (Figure S1G, 1H), and PCR revealed the efficacy of gene silencing in CSps (data not shown). Collagen expression after coculture of siRNA CSp-conditioned media with fibroblasts was similar to that in normally-cultured fibroblasts (Figure S1I); thus, the expression of these two MMPs plays a critical role in collagen degradation after CSp injection. It seems likely that differential *in vivo* expression of MMP2 and MMP9 underlies the differences in tissue pathology and remodeling.

### Phenotypic changes in the small MI group

As shown in Figure 1D, CSps potently decrease infarct size when administered in the acute MI setting. We therefore asked if the CSp-related morphological benefits and changes in fibrosis are entirely a consequence of infarct size reduction. If CSps have an acute cardioprotective effect, as seems likely given their anti-apoptotic effects [13], [17], [25], myocardial architecture would be better-preserved, even if CSps had no long-term regenerative benefit or primary inhibitory effects on fibrosis. The fourth group with the initially smaller MI served to test this hypothesis. An infarct size similar to that in the CSp group was achieved by more distal ligation of the LAD, without cell therapy (Figure S2). Although infarct size in the “Small MI” group ended up indistinguishable from that in the MI+CSp group (Figures S2A–I), we detected less densely-populated cardiomyocytes (Figures S3A–E), lower cardiomyocyte proliferation (Figures S3F–I), lower capillary density (Figures S4A–D) and more collagen (Figures S5A–C) than in the CSp-treated myocardium. Thus, CSps exert mechanistically-distinct regenerative effects above and beyond the consequences of simple cardioprotection and infarct size reduction.

## Discussion

The novel aspects of the present study are as follows: 1) we demonstrated for the first time tissue-level changes underlying the profound functional benefits of cardiospheres, and we provided a pathophysiologic rationale for the mechanism of the attenuated remodeling. 2) More specifically, we showed that the gross changes of MI mass reduction and LV functional preservation in the CSp-treated group were accompanied by attenuated global cardiomyocyte hypertrophy. 3) CSp injection increased cardiomyocyte proliferation in the border zone, and had a similar but lesser effect in remote myocardium. 4) We showed *in vivo* that CSps reduced regional collagen deposition and, *in vitro*, that this antifibrotic effect is partially mediated by secreted MMPs. 5) We demonstrated increased angiogenesis post-CSp transplantation *in vivo* and *ex vivo* in aortic rings. 6) Finally, the beneficial effects of cardiospheres were notable even when compared to those in animals with smaller MIs, indicating an anti-remodeling effect of the CSps beyond a possible acute reduction of infarct size. Collectively, our data demonstrate that CSps play a pivotal role in the attenuation of ventricular remodeling via a multifaceted mechanistic cascade involving enhanced cardiomyocyte proliferation, attenuated cardiomyocyte hypertrophy, activation of MMPs and the secretion of angiogenic paracrine factors.

The extracellular matrix is the “skeleton” of the myocardium which controls its geometry and architecture [41]. It provides a scaffold for myocytes, fibroblasts, endothelial cells and the vasculature to align and build a functional syncytium. Fibroblast recruitment, fibroblast proliferation, and collagen deposition combine to form a scar soon after the insult, leading to myocardial stiffness and dysfunction [30], [36], [42]. Increased stiffness favors apoptosis of stem cells and decreases the potential differentiation of stem cells into cardiomyocytes [43]–[46]. Cardiospheres decreased post-MI fibrosis, at least partly by MMP activation. The specific pattern of activation of MMPs *in vivo* consisted of increased ICTP levels and reduced collagen production and tissue deposition, while the reduction of collagen in the coculture experiments indicates an antifibrotic effect of transplanted cardiospheres. However, the Tgfb and the epithelial mesenchymal transition cascades also play important roles in cardiac fibrosis; potential interaction of CSps with these pathways cannot be excluded and indeed merits further study [47].

The antifibrotic effect may be causally related to the observed enhancement of microvessel density. Excess collagen deposition is an impediment to endothelial progenitor cell migration and proliferation and, as a consequence, to capillary formation [48]. The inadequacy of the vasculature to adapt to the infarcted myocardium's metabolic demands is also a critical compensatory mechanism [45], [49]. CSp transplantation triggers release of endogenous growth factors, including VEGF, HGF, and IGF, which are able to promote neoangiogenesis, restore blood flow, and increase cardiac function [25], [26]. Likewise, CSp injection enhanced vascularity in both the peri-infarct and remote zone. Following MI, complex interactions among cardiac fibroblasts, myofibroblasts, myocytes, endothelial cells, and ECM constituents modulate and define the ventricular functional repertoire [30]. These control the formation of muscle tensile strength essential for efficient systolic and diastolic function. The peri-infarct zone is the one exposed to the greatest stress, so it is possible that the increased vessel density in the post-CSp transplanted myocardium supports the function of this vulnerable region, abrogating further expansion of the infarct and limiting global remodeling [49]–[51].

Although the enhanced vessel number can be attributed to the cardioprotective effects of CSps, their paracrine actions to form microvessels were underscored here by the *ex vivo* angiogenesis assay where an aortic ring treated with CSp-conditioned media induced sprout formation. CSp engraftment in vivo is evanescent [26] (<1% of transplanted cells can be detected 3 weeks post-injection), but this time course appears to suffice to induce significant neovascularization.

In addition, another effect of the transplanted CSps is the stimulation of resident cells to proliferate [52]. Here, we found that CSp transplantation was followed by almost three-fold upregulation of the cardiomyocyte renewal in the peri-infarct zone. The release of growth factors, which has been detected in peri-infarct and remote myocardium post-CSp transplantation [26], presumably enhances cell proliferation and cell survival, although direct cell-cell interactions may also play a role [53]. The observed reduction in cardiomyocyte cross-sectional area is consistent with the increase in cardiomyocyte proliferation. Synergistically, alleviation of wall stress as a consequence of attenuated remodeling would further contribute to the observed antihypertrophic effect. Hypertrophy post-MI is posited to diminish oxygen consumption [48], but is associated with increased risk of heart failure, malignant arrhythmia and metabolic activity [48]–[51]. Thus, the structural sequelae of CSp transplantation would be logically predicted to have beneficial outcomes.

The beneficial effects of CSp transplantation were evident even when compared to the smaller MI group. Post-MI, infarct expansion takes place within hours and leads to wall thinning, ventricular dilatation, increases in diastolic and systolic wall stresses, and deterioration of ventricular performance [54]. Cardiomyocyte hypertrophy, decreased vessel density and neurohormonal stimulation further aggravate early myocardial remodeling. Although the infarct size in the small MI group was comparable to that in the CSp-treated group, greater cardiomyocyte hypertrophy, worse LV dilatation and higher collagen deposition were observed in the former group, highlighting the anti-remodeling effect of CSps. Thus, CSps do not exert structural and functional benefits solely because of acute infarct size reduction.

Long-term engraftment of CSps is not a prerequisite for durable LV functional improvement [25], [26]. The present study is the first to dissect the potential tissue-level structural mechanisms whereby cardiospheres exert these beneficial effects. Cardiosphere-derived cells (CDCs) exert greater functional benefits compared to other clinically-tested cell types [17]. Cardiospheres, in turn, are superior to CDCs when both are injected intramyocardially post-MI [13], [18]. Here, a relatively low number of cardiospheres was able to interrupt post-MI adverse structural remodeling, highlighting the significance of CSps as a potential therapeutic tool for ischemic cardiac disease. So far, the increased size of our multicellular clusters has been thought to preclude intracoronary delivery, but percutaneous intramyocardial injections may be a clinically-realistic option [19], [55], [56]. It is also notable that CSps injected into the coronary arteries, although embolic, can undergo extravasation [57], raising the possibility that intracoronary delivery may be viable over a carefully-determined dosage range.

### Limitations

The aim of the current study was to test the hypothesis that CSp transplantation alters myocardial architecture post-MI; thus, an acute MI model was used. We are also interested in extending our previous results that CSps may reverse established remodeling in a chronic MI model [31], but this requires separate study. In addition, the detailed *in vivo* time course of MMP remains to be defined, and such studies could help to differentiate the antifibrotic and collagenolytic effects of CSps reported here. We did, though, measure ICTP levels contemporaneously with MMPs (7 days), a finding which supports enhanced collagenolysis. Thirdly, longer-term morphological evaluation could be critical from a clinical standpoint. In this regard, we have found durable functional benefits of injected cardiospheres [26] up to six months after follow up in a similar model, rendering unlikely the possibility that CSps' benefits are transient.

## Conclusions

In conclusion, we find that cardiospheres decrease interstitial collagen deposition and wall stiffness, and produce profound capillary neoformation, supplying new cardiomyocytes with a supportive microenvironment. The fact that CSps impact on several diverse, but potentially synergistic, repair processes hints at molecular orchestration by microRNAs [58]. Intriguingly, we find that CDCs secrete exosomes rich in microRNAs [59], [60]. The present findings provide a rationale for the attenuated cardiac remodeling seen with transplantation of cardiospheres and CDCs, a principle central to their functional benefits in ischemic cardiac disease [26], [61].

## Supporting Information

Figure S1 ***In vitro*** **collagenolytic activity of CSps.** (A,B) Confocal images of immunostained CSps for MMP2 and MMP9 expression respectively. (C) Coculture systems for serum free and conditioned media treated dermal fibroblasts. (D) Gel zymography shows enhanced MMP2 and MMP9 activity 24 h after coculture of CSp conditioned media and fibroblasts compared to fibroblasts alone. (E) Quantification of the MMP activity in the supernatant of the coculture system, the conditioned media alone and the fibroblasts alone. (F) Immunoblotting for col1agen expression by the fibroblasts under serum free and conditioned media treatment revealed showing reduced collagen 24 h post conditioned media treatment. (G,H) Gel zymography shows reduced CSp conditioned media MMP2 and MMP9 activity post siRNA treatment, which inhibited the collagen degradation verified by immunoblotting 24 h post coculture (I).
(TIF)

Figure S2 **Tissue Morphology and LV Functional Evaluation.** (A) LV morphometry evaluation in all three groups studied. (B,E). The small MI group had an infarct mass similar to the treated group, but a thinner infarct wall thickness. (C,D,F) Viable mass, scar size and septal thickness were similar between small MI and MI+CSp groups. (G) A significantly higher EF was detected at baseline in the small MI group compared to both placebo and CSp treated, but progressively deteriorated up to 21 days after evaluation. (H) Significant dilation of the LV evaluated with the LVEDD was also present at day 7 and day 21 in the small MI group compared to the CSp treated but, (I) no difference was measured between the two groups as far as the LVPWD is concerned. Data are mean±SD. ¶ p<0.05 control vs MI+CSp, * p<0.05 vs. sham, # p<0.05 vs. small MI.
(TIF)

Figure S3 **Cardiac tissue structure overview.** (A–E) Small MI group exerted significant hypertrophy compared to the sham group which was accompanied by significant reduction in the cell nuclei number compared to the sham and the treated group. (F–I) Quantification of cardiomyocyte proliferation in both peri-infarct and remote regions 7 days post MI and treatment. A trend toward enhanced Ki67+ cardiomyocytes without reaching significance compared to the control was observed in the small MI group. Data are mean±SD. ¶ p<0.05 control vs. MI +CSp, * p<0.05 vs. sham, # p<0.05 vs. small MI.
(TIF)

Figure S4 **Cardiospheres stimulate angiogenesis.** (A.D) Quantification of the capillary density with vWf staining and of the vessels' density with sma staining in both border and remote regions. CSp treatment triggered significant neoangiogenesis even compared to the small MI group reflecting bona fide effect of CSpsin the vessel formation. Data are mean±SD. ¶ p<0.05 control vs. MI+CSp, * p<0.05 vs. sham, # p<0.05 vs. small MI.
(TIF)

Figure S5 **Collagen quantification.** (A–C) Collagen content quantification by Picrosirius Red staining revealed a higher deposition in the LV on the small MI group associated with significantly increased content especially in the border zone compared to the CSp treated group. Data are mean±SD. ¶ p<0.05 control vs. MI+CSp, * p<0.05 vs. sham, # p<0.05 vs. small MI.
(TIF)
