# Supplementary figures and images for: Angiogenesis, Cardiomyocyte Proliferation and Anti-Fibrotic Effects Underlie Structural Preservation Post-Infarction by Intramyocardially-Injected Cardiospheres
Source: PLoS One. 2014 Feb 18;9(2):e88590. doi: 10.1371/journal.pone.0088590 (PMC3928273; doi:10.1371/journal.pone.0088590)

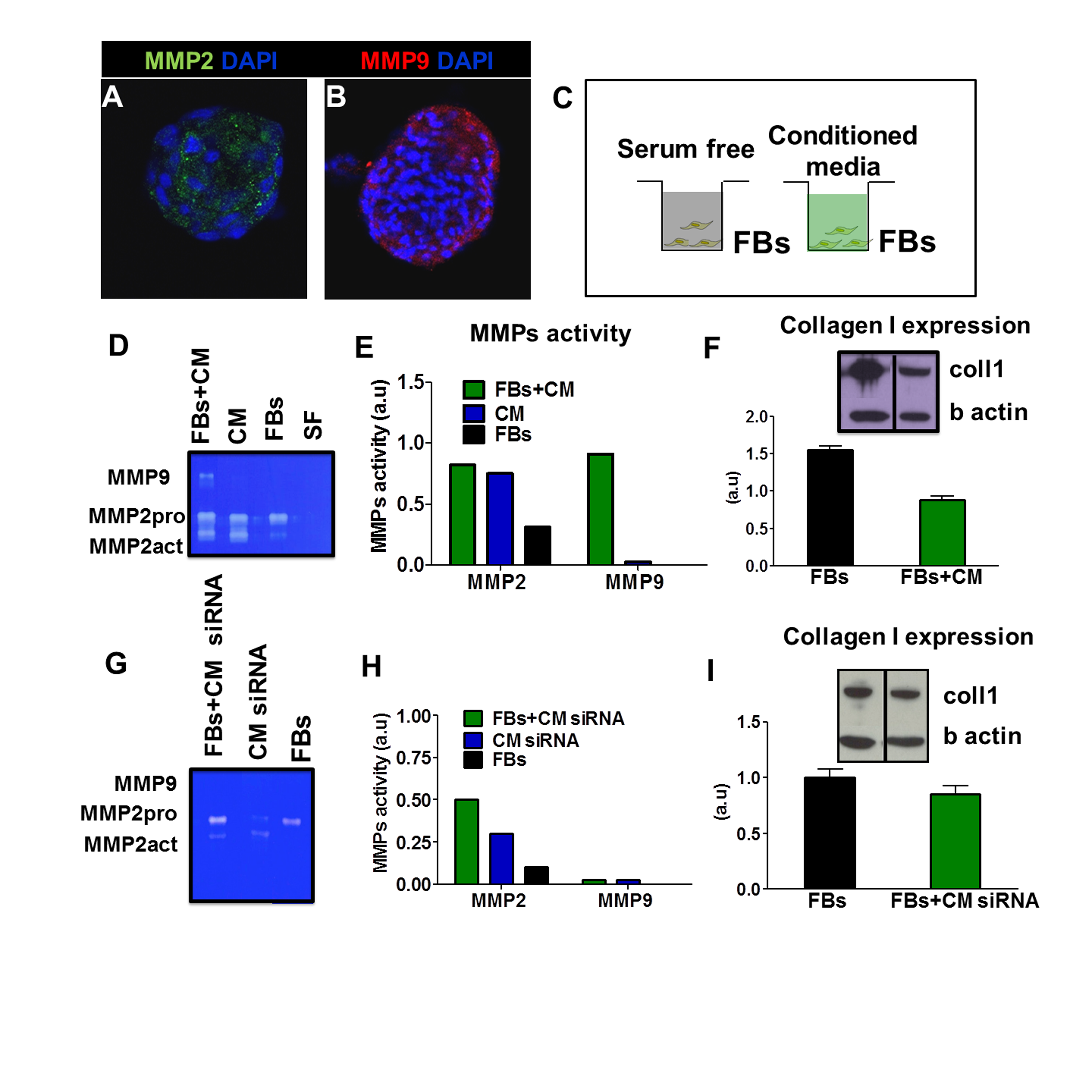

Supplement: Figure S1 — In vitro collagenolytic activity of CSps. (A,B) Confocal images of immunostained CSps for MMP2 and MMP9 expression respectively. (C) Coculture systems for serum free and conditioned media treated dermal fibroblasts. (D) Gel zymography shows enhanced MMP2 and MMP9 activity 24 h after coculture of CSp conditioned media and fibroblasts compared to fibroblasts alone. (E) Quantification of the MMP activity in the supernatant of the coculture system, the conditioned media alone and the fibroblasts alone. (F) Immunoblotting for col1agen expression by the fibroblasts under serum free and conditioned media treatment revealed showing reduced collagen 24 h post conditioned media treatment. (G,H) Gel zymography shows reduced CSp conditioned media MMP2 and MMP9 activity post siRNA treatment, which inhibited the collagen degradation verified by immunoblotting 24 h post coculture (I). (TIF) [file pone.0088590.s001.tif]

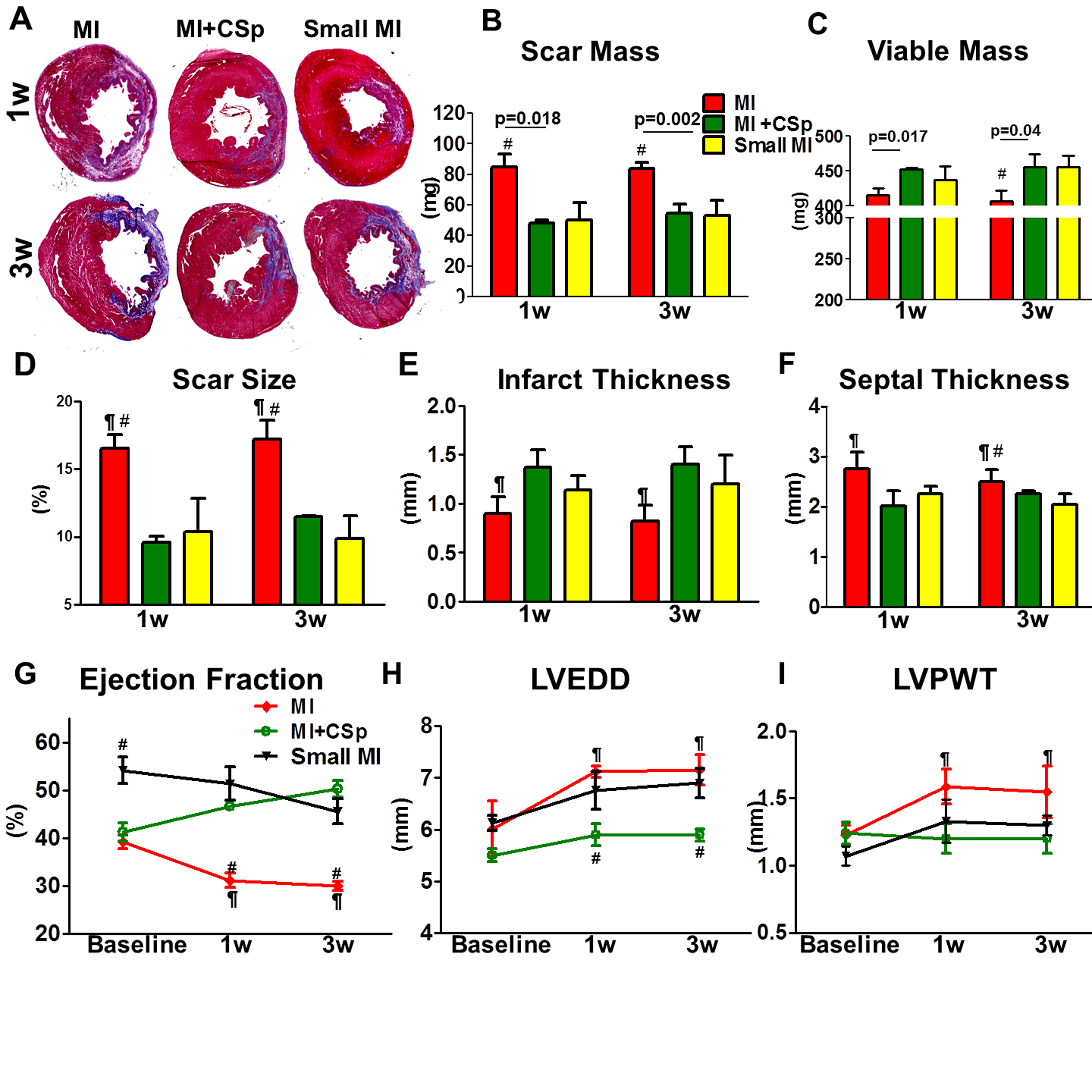

Supplement: Figure S2 — Tissue Morphology and LV Functional Evaluation. (A) LV morphometry evaluation in all three groups studied. (B,E). The small MI group had an infarct mass similar to the treated group, but a thinner infarct wall thickness. (C,D,F) Viable mass, scar size and septal thickness were similar between small MI and MI+CSp groups. (G) A significantly higher EF was detected at baseline in the small MI group compared to both placebo and CSp treated, but progressively deteriorated up to 21 days after evaluation. (H) Significant dilation of the LV evaluated with the LVEDD was also present at day 7 and day 21 in the small MI group compared to the CSp treated but, (I) no difference was measured between the two groups as far as the LVPWD is concerned. Data are mean±SD. ¶ p<0.05 control vs MI+CSp, * p<0.05 vs. sham, # p<0.05 vs. small MI. (TIF) [file pone.0088590.s002.tif]

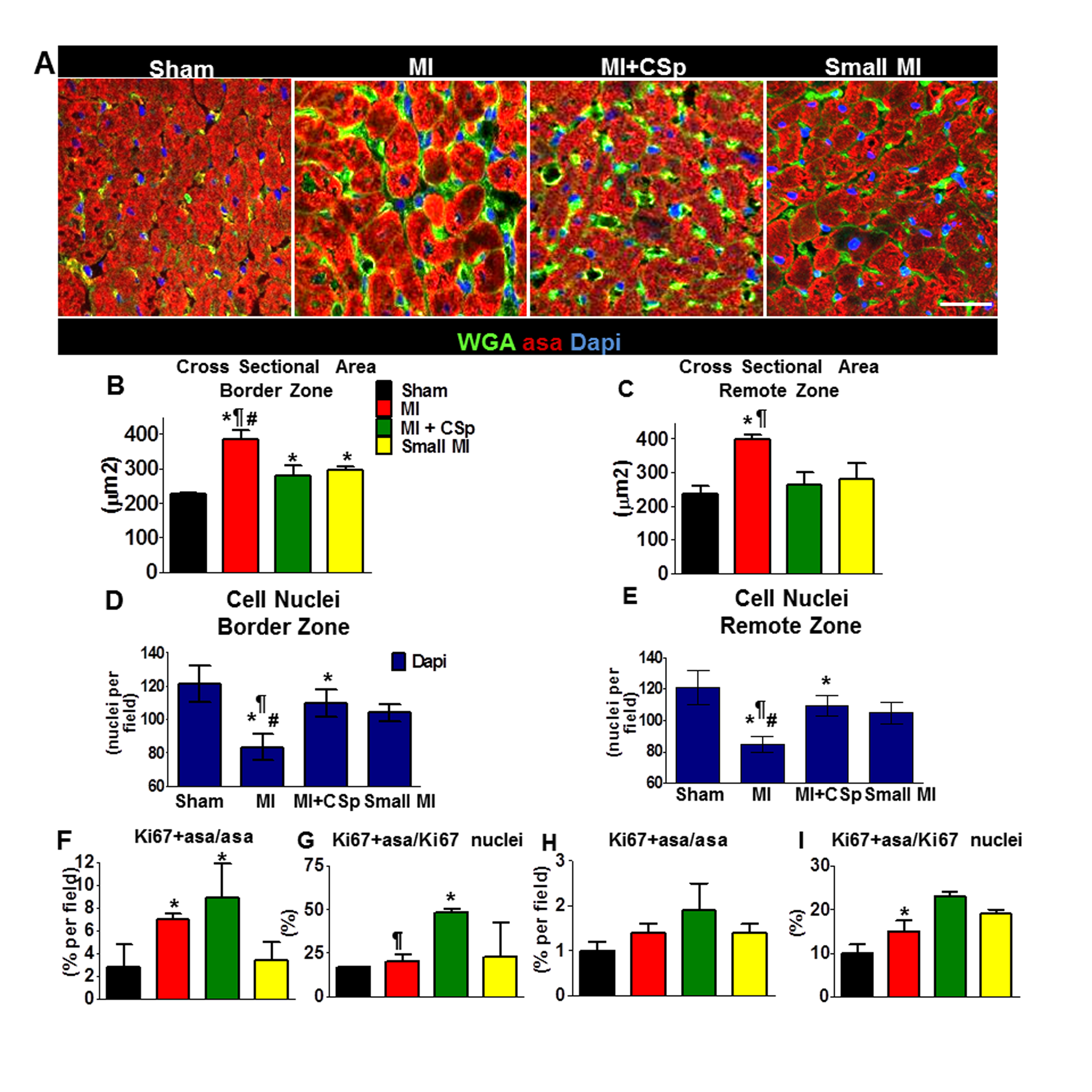

Supplement: Figure S3 — Cardiac tissue structure overview. (A–E) Small MI group exerted significant hypertrophy compared to the sham group which was accompanied by significant reduction in the cell nuclei number compared to the sham and the treated group. (F–I) Quantification of cardiomyocyte proliferation in both peri-infarct and remote regions 7 days post MI and treatment. A trend toward enhanced Ki67+ cardiomyocytes without reaching significance compared to the control was observed in the small MI group. Data are mean±SD. ¶ p<0.05 control vs. MI +CSp, * p<0.05 vs. sham, # p<0.05 vs. small MI. (TIF) [file pone.0088590.s003.tif]

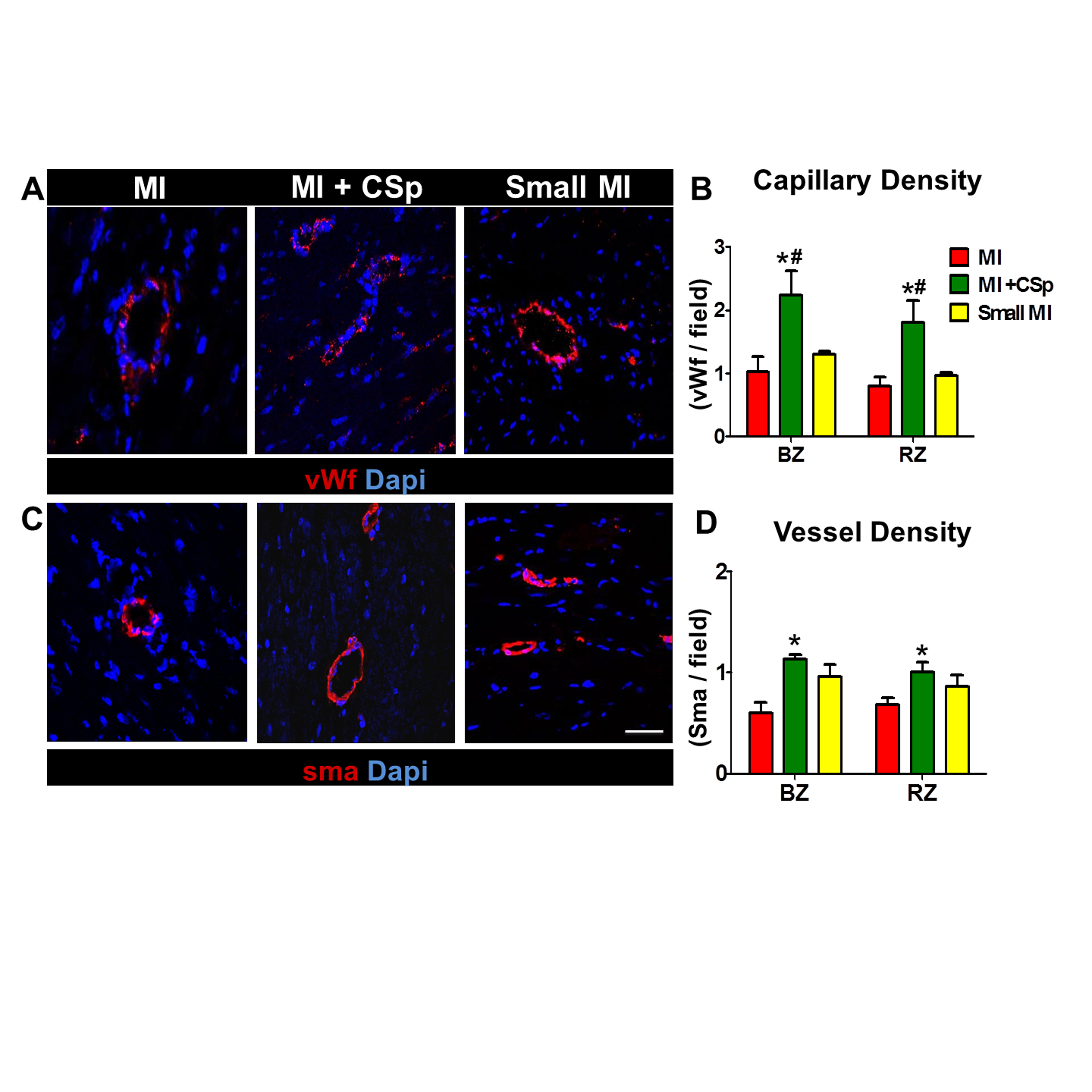

Supplement: Figure S4 — Cardiospheres stimulate angiogenesis. (A.D) Quantification of the capillary density with vWf staining and of the vessels' density with sma staining in both border and remote regions. CSp treatment triggered significant neoangiogenesis even compared to the small MI group reflecting bona fide effect of CSpsin the vessel formation. Data are mean±SD. ¶ p<0.05 control vs. MI+CSp, * p<0.05 vs. sham, # p<0.05 vs. small MI. (TIF) [file pone.0088590.s004.tif]

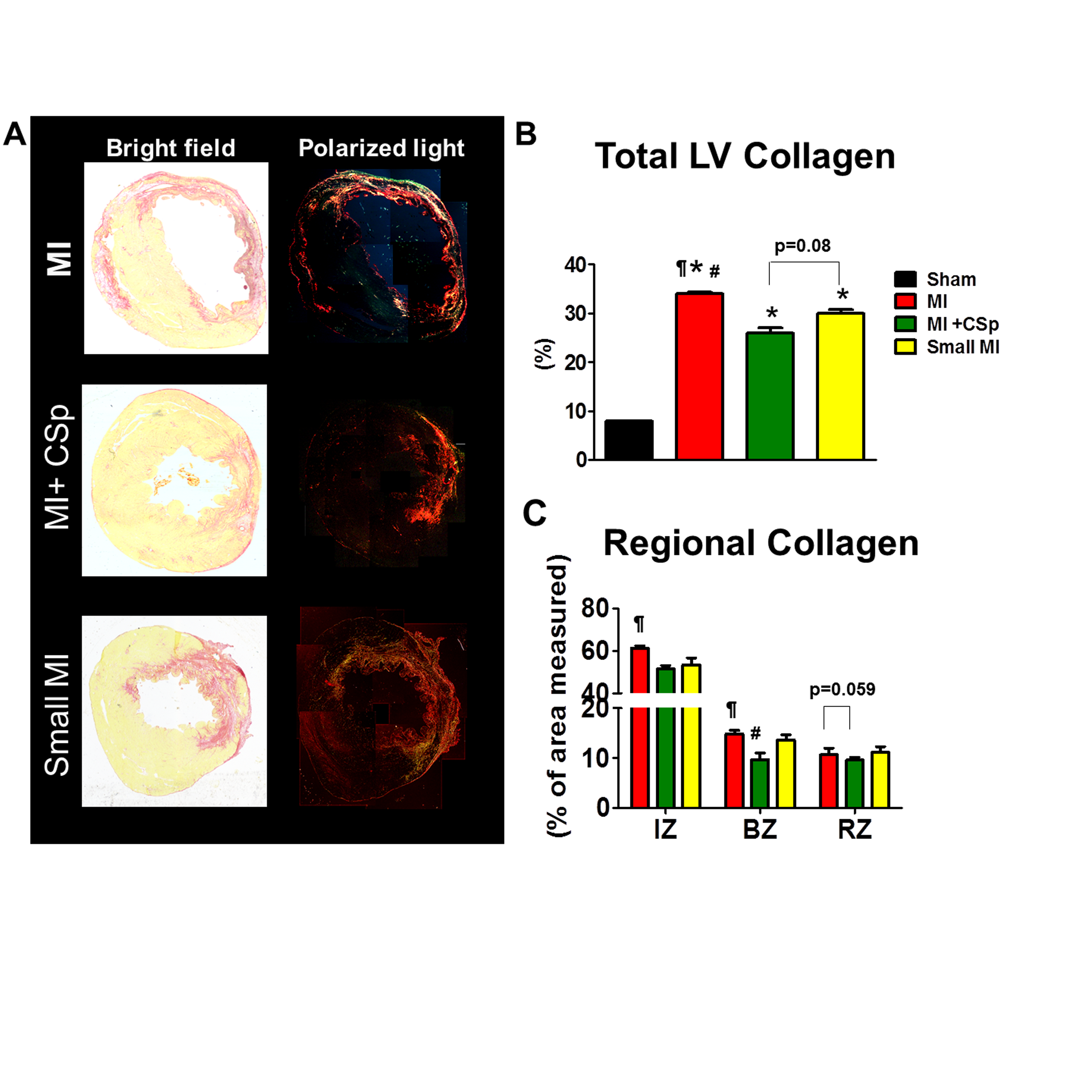

Supplement: Figure S5 — Collagen quantification. (A–C) Collagen content quantification by Picrosirius Red staining revealed a higher deposition in the LV on the small MI group associated with significantly increased content especially in the border zone compared to the CSp treated group. Data are mean±SD. ¶ p<0.05 control vs. MI+CSp, * p<0.05 vs. sham, # p<0.05 vs. small MI. (TIF) [file pone.0088590.s005.tif]
